# Neural substrates underlying delusions in schizophrenia

**DOI:** 10.1038/srep33857

**Published:** 2016-09-21

**Authors:** Jiajia Zhu, Chuanjun Zhuo, Feng Liu, Lixue Xu, Chunshui Yu

**Affiliations:** 1 Department of Radiology and Tianjin Key Laboratory of Functional Imaging, Tianjin Medical University General Hospital, Tianjin 300052, China; 2 Department of Psychiatry Functional Neuroimaging Laboratory, Tianjin Mental Health Center, Tianjin Anding Hospital, Tianjin 300070, China

## Abstract

Delusions are cardinal positive symptoms in schizophrenia; however, the neural substrates of delusions remain unknown. In the present study, we investigated the neural correlates of delusions in schizophrenia using multi-modal magnetic resonance imaging (MRI) techniques. Diffusion, structural and perfusion MRIs were performed in 19 schizophrenia patients with severe delusions, 30 patients without delusions and 30 healthy controls. Fractional anisotropy (FA), gray matter volume (GMV) and cerebral blood flow (CBF) were voxel-wisely compared among the three groups. Although patients without delusions exhibited decreased FA in white matter regions and decreased GMV in gray matter regions relative to controls, patients with severe delusions demonstrated comparable FA in all of these white matter regions and similar GMV in most of these gray matter regions. Both patient subgroups had less GMV in the amygdala and anterior cingulate cortex than controls. Although two patient subgroups showed consistent CBF changes relative to controls, only CBF in the anterior cingulate cortex was lower in patients with severe delusions than in patients without delusions. These findings suggest that schizophrenia patients with severe delusions have relatively normal structural integrity. Importantly, the excessively reduced perfusion in the anterior cingulate cortex may be associated with the development of delusions in schizophrenia.

Schizophrenia manifests as a complex composition of many symptoms, including delusions, hallucinations, disorganization, avolition, apathy, and cognitive decline^1^,^2^. Each symptom shows variable degrees of expression in individual patients and possibly has unique neuropathology and etiology in schizophrenia. It has been suggested that clinical subtypes of schizophrenia have distinct genetic antecedents^3^ and are associated with different brain imaging changes^4^. The inconsistencies across studies may arise from all-inclusive, unpartitioned clinical phenotypes. Identification of the relevant features specific to a certain schizophrenic symptom is important for precision clinical diagnosis, treatment, and research. Delusions are one of the cardinal positive symptoms of schizophrenia^5^ and affects more than 70% of schizophrenia patients^6^. It has been shown that delusions are associated with specific behavioral deficits, such as defective verbal self-monitoring^7^,^8^ and low cognitive insight^9^. However, the neural mechanisms of delusions in schizophrenia remain largely unknown.

Delusions probably arise from the brain’s attempts to integrate erroneous perceptions and disorganized neural processes^10^. To identify neural substrates of delusions, multiple diffusion tensor imaging (DTI) and structural magnetic resonance imaging (MRI) studies have attempted to identify correlations between structural impairments and delusions in schizophrenia (Supplementary Table S1 and S2). However, both positive^11^,^12^,^13^,^14^,^15^,^16^,^17^,^18^,^19^,^20^ and negative^21^,^22^,^23^,^24^,^25^,^26^,^27^,^28^,^29^,^30^,^31^,^32^,^33^ correlations have been reported. As a measure of cerebral perfusion, both positive and negative correlations between regional cerebral blood flow (CBF) and delusions have been found in schizophrenia^34^. These discrepant findings prevent us from drawing a conclusion about the association between structural/functional impairments and delusions in schizophrenia.

Most of the previous studies on the neural substrates of delusions in schizophrenia have been based on a single imaging modality, which has prevented us from establishing a comprehensive understanding on the question. However, the developments of MRI imaging and processing techniques now provide us an opportunity to better characterize brain structural and functional changes in brain disorders. The DTI theory assumes that the diffusion of water molecules occurs in a free and unrestricted environment with a perfect Gaussian probability distribution^35^, which is not valid in biological tissues where water molecules often display non-Gaussian diffusion due to the presence of barriers such as cell membranes and organelles^36^. Diffusion kurtosis imaging (DKI) has been proposed to simultaneously characterize Gaussian and non-Gaussian diffusion^37^,^38^,^39^,^40^,^41^, which can make a more accurate estimation of fractional anisotropy (FA) than the DTI technique^40^,^42^. Moreover, tract-based spatial statistics (TBSS)^43^ is proposed as a more reasonable method to compare FA differences between groups because it can overcome the misalignment across subjects in the traditional voxel-based analysis. The diffeomorphic anatomical registration through the exponentiated Lie algebra (DARTEL) technique largely improves the precision of anatomical registration^44^, which is especially suitable for gray matter volume (GMV) analysis. The three-dimensional pseudo-continuous arterial spin labeling (3D-pcASL) MRI technique is becoming the best choice for noninvasive CBF measurement because of its high efficiency, multi-slice capability, and relative ease of implementation^45^. Compared with the blood oxygen-level-dependent (BOLD) functional MRI, the 3D-pcASL is less affected by susceptibility artifacts, and the CBF has a more definite physiological implication. Joint use of the three MRI techniques may improve our understanding of the neural substrates of delusions in schizophrenia.

In this study, we used FA to assess white matter integrity, GMV to assess gray matter structural properties, and CBF to assess gray matter functional properties. By voxel-wise comparisons of the FA, GMV and CBF among schizophrenia patients with severe delusions, patients without delusions and healthy controls, we aimed to identify the structural and functional substrates of delusions in schizophrenia.

## Materials and Methods

### Participants

The Ethics Committee of Tianjin Medical University General Hospital approved this study, and all participants provided written informed consent prior to study participation. The methods were carried out in accordance with the approved guidelines. This study included 19 schizophrenia patients with severe delusions, 30 schizophrenia patients without delusions and 30 healthy controls. The patients were recruited from inpatient and outpatient facilities in Tianjin Mental Health Center and Tianjin Anning Hospital, and the healthy controls were recruited from local community via advertisements. Diagnosis of schizophrenia was determined by consensus of two experienced psychiatrists using the Structured Clinical Interview for the DSM-IV Axis I Disorder, Patient Edition (SCID-P). All healthy controls were screened using the non-patient edition of the SCID (SCID-NP) to confirm an absence of psychiatric illnesses. The inclusion criteria were age (20–60 years) and right-handedness. The exclusion criteria for all participants were MRI contraindications, the presence of a systemic medical illness (e.g., cardiovascular disease, diabetes mellitus) or central nervous system disorder (e.g., epilepsy) that would affect the study results, a history of head trauma (e.g., hemorrhage), or substance (e.g., hypnotics, alcohol) abuse within the past 3 months or a lifetime history of substance dependence. Additional exclusion criteria for the healthy controls included a history of psychiatric disease and first-degree relatives with a history of psychotic episodes.

According to DSM-IV schizophrenia subtypes, the 19 patients with severe delusions were further subdivided into paranoid (n = 14), disorganized (n = 1), undifferentiated (n = 3), and residual (n = 1) subtypes; and the 30 patients without delusions were subdivided into paranoid (n = 11), disorganized (n = 1), catatonic (n = 1), and undifferentiated (n = 17) subtypes. Only 2 patients with severe delusions were first-episode schizophrenia, and the rest of patients were chronic schizophrenia. Only four patients in the severe delusion group had never received any medications, and the rest of patients were receiving atypical antipsychotic medications when performing MRI examinations. Demographic and clinical data for these subjects are shown in Table 1.

Delusion severity was assessed by the P1 sub-scores from the Positive and Negative Syndrome Scale (PANSS)^46^. No delusion was defined as P1 = 1, and severe delusions were defined as P1 ≥ 6. Only schizophrenia patients with severe delusions were selected because these patients are thought to exhibit the most typical and definite delusion symptoms. The subtypes of delusions^47^ in these patients are shown in (Table S3.

### MRI data acquisition

MRI examinations were performed using a 3.0-Tesla MR system (Discovery MR750, General Electric, Milwaukee, WI, USA). Tight but comfortable foam padding was used to minimize head motion, and earplugs were used to reduce scanner noise. DKI data were acquired by a spin-echo single-shot echo planar imaging sequence with the following parameters^39^: repetition time (TR) = 5800 ms; echo time (TE) = 77 ms; matrix = 128 × 128; field of view (FOV) = 256 mm × 256 mm; in-plane resolution = 2 mm × 2 mm; slice thickness = 3 mm without gap; 48 axial slices; 25 encoding diffusion directions with two values of b (b = 1000 and 2000 s/mm^2^) for each direction; 10 non-diffusion-weighted images (b = 0 s/mm^2^); and acquisition time = 354 s. Sagittal 3D T1-weighted images were acquired by a brain volume sequence (TR = 8.2 ms; TE = 3.2 ms; inversion time = 450 ms; flip angle = 12°; FOV = 256 mm × 256 mm; matrix = 256 × 256; slice thickness = 1 mm, no gap; 188 sagittal slices; and acquisition time = 250 s). The resting-state perfusion imaging was performed using a pcASL sequence with a 3D fast spin-echo acquisition and background suppression (TR = 4886 ms, TE = 10.5 ms, post-label delay = 2025 ms, spiral in readout of eight arms with 512 sample points; flip angle = 111°; FOV = 240 mm × 240 mm; reconstruction matrix = 128 × 128; slice thickness = 4 mm, no gap; 40 axial slices; number of excitation = 3; 1.9 mm × 1.9 mm in-plane resolution; and acquisition time = 284 s). The label and control whole-brain volumes required 8 TRs, respectively. A total of three pairs of label and control volumes were acquired. All images were visually inspected to ensure that only images without visible artifacts were included in subsequent analyses.

### FA calculation and analysis

Eddy current-induced distortion and motion artifacts in the DKI dataset were corrected by applying affine alignment of each diffusion-weighted image to the b = 0 image using the FMRIB Software Library (FSL 4.0, http://www.fmrib.ox.ac.uk/fsl). After skull-stripping, Diffusional Kurtosis Estimator software (www.nitrc.org/projects/dke) was implemented to calculate the diffusion and kurtosis tensors using constrained linear least squares-quadratic programming (CLLS-QP) algorithm^48^. The FA parametric map was generated from DKI fitting, which requires at least two non-zero b values in more than 15 independent directions.

TBSS was used to compare the FA differences in brain white matter among the three groups. All subjects’ FA maps were aligned to a template of the averaged FA map (FMRIB-58) in Montreal Neurological Institute (MNI) space using a non-linear registration algorithm. After transformation into MNI space, a mean FA map was created and thinned to generate a mean FA skeleton of the white matter. Each subject’s FA map was then projected onto the skeleton by filling the mean FA skeleton with FA values from the nearest relevant tract center by searching perpendicular to the local skeleton structure for the maximum FA value.

### GMV calculation

The VBM8 toolbox (http://dbm.neuro.uni-jena.de/vbm.html) was used to calculate GMV. Structural MRI images were segmented into gray matter, white matter and cerebrospinal fluid using the standard segmentation model. After an initial affine registration of the gray matter concentration map into MNI space, the gray matter concentration images were nonlinearly warped using the DARTEL technique and then resampled to a voxel size of 1.5 mm × 1.5 mm × 1.5 mm. The GMV was obtained by multiplying the gray matter concentration map by the non-linear determinants that were derived from the spatial normalization step. Finally, the GMV images were smoothed using a Gaussian kernel of 6 mm × 6 mm × 6 mm full-width at half maximum (FWHM).

### CBF calculation

An ASL difference image was calculated using a single-compartment model^49^ after the subtraction of the label image from the control image. The three ASL difference images were averaged to calculate CBF maps in combination with the proton-density-weighted reference image^50^. SPM8 software was used to normalized the CBF images to the MNI space using the following steps: (1) the naive ASL difference images of healthy controls were non-linearly normalized to the MNI space and then averaged to generate a study-specific standard ASL template; (2) the native ASL difference image of each subject was non-linearly co-registered to the study-specific standard ASL template; and (3) the CBF image of each subject was written into the MNI space using the deformation parameter derived from the prior step and was resampled to a voxel size of 2 mm × 2 mm × 2 mm. Then, each co-registered CBF map was removed of non-brain tissue and spatially smoothed with a Gaussian kernel of 8 mm × 8 mm × 8 mm FWHM. A previous study has demonstrated that normalized CBF is more reasonable for inter-group comparison than absolute CBF, because the former can reduce the effect of inter-subject variations in global CBF^51^. Therefore, we normalized the CBF of each voxel by dividing the mean CBF of the whole brain.

### Statistical analysis

For TBSS analysis of the FA, the threshold-free cluster enhancement (TFCE)^52^ option in the permutation-testing tool (permutations = 5000) in the FSL software was used to test statistical significance. A family-wise error (FWE) method was used to correct for multiple comparisons with a significance threshold of *P* < 0.05. For voxel-based analyses of GMV and CBF, the parametric test in the SPM8 package was used to test statistical significance. Multiple comparisons were corrected using a false discovery rate (FDR) method with a significance threshold of *P* < 0.05. A voxel-wise one-way analysis of covariance (ANCOVA) was used to test the FA, GMV and CBF differences across the three groups with age and gender as nuisance covariates. The FA analysis was restricted to the mean white matter skeleton while the GMV and CBF analyses were restricted to the gray matter mask. If a measure revealed a significant difference across groups in a cluster, the mean value of all voxels within this cluster was extracted for region of interest (ROI)-based *post hoc* analyses. For each cluster having a significant group difference in any of the three measures, a general linear model was used to compare the differences in FA, GMV or CBF between every two of the three groups while controlling for age and gender (*P* < 0.05/3 = 0.017, Bonferroni-corrected). In addition, we also repeated the voxel-wise ANCOVA without age covariate.

To test the possible effects of antipsychotic medications and duration of illness on our results, we performed ROI-based comparisons in FA, GMV and CBF between schizophrenia patients with severe delusions and without delusions before and after controlling for dosage of chlorpromazine equivalents and duration of illness. To test the effects of other symptoms, we also took the score of PANSS except delusions as a covariate in the ROI-based comparison analyses between the two patient groups.

To show the inter-group difference in each measure more intuitively, we directly performed voxel-wise whole brain comparisons in FA, GMV and CBF between every two of the three groups using a two-sample *t*-test while controlling for age and gender. Multiple comparisons were corrected using the same methods with the voxel-wise ANCOVA, and the significance threshold was set at *P* < 0.05.

## Results

### Demographic and clinical characteristics

Table 1 contains demographic and clinical information for the study sample. We included 19 patients with severe delusions (7 females; age: 34.6 ± 9.4 years), 30 patients with no delusions (16 females; age: 35.9 ± 9.2 years) and 30 healthy controls (15 females; age: 35.3 ± 9.3 years). There were no significant differences in gender (chi-square test, *χ*^*2*^ = 1.337, *P* = 0.512) and age (one-way ANOVA, *F* = 0.112, *P* = 0.894) among the three groups. There were also no significant differences in antipsychotic dosages (two sample t-test, *t* = 0.709, *P* = 0.482) and durations of illness (two sample t-test, *t* = −1.741, *P* = 0.148) between the two patient subgroups.

### FA differences across groups

**Figure 1:**
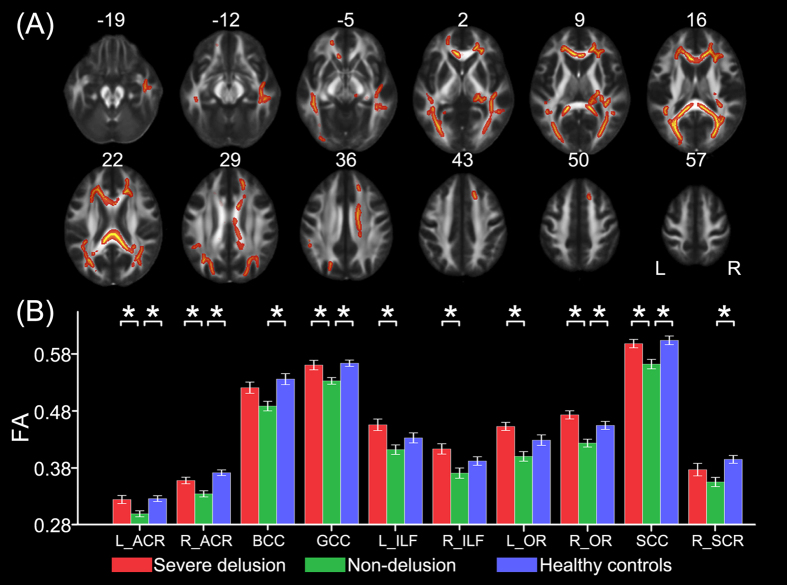
FA differences across groups. TBSS analysis shows white matter tracts with FA differences (*P* < 0.05, FWE corrected) across the three groups (**A**). A *post hoc* ROI analysis shows the pair-wise comparisons (*P* < 0.05, corrected) (**B**). Error bars indicate the standard error of the mean. *P < 0.05, corrected. Abbreviations: ACR, anterior corona radiata; BCC, body of corpus callosum; FA, fractional anisotropy; GCC, genu of corpus callosum; ILF, inferior longitudinal fasciculus; L, left; OR, optic radiation; R, right; SCC, splenium of corpus callosum; SCR, superior corona radiata.

One-way ANCOVA identified 10 white matter tracts that showed significant differences (*P* < 0.05, FWE corrected) in FA among the three groups including the genu (GCC), the body (BCC), the splenium (SCC) of the corpus callosum, the bilateral anterior corona radiata (ACR), the bilateral inferior longitudinal fasciculus (ILF), the bilateral optic radiation (OR), and the right superior corona radiata (SCR) (Fig. 1A). The *post hoc* analyses revealed that schizophrenia patients without delusions showed significantly reduced FA in the GCC, the SCC, the bilateral ACR, and the right OR compared to both patients with severe delusions and healthy controls, in the BCC and the right SCR compared to healthy controls, and in the bilateral ILF and the left OR compared to patients with severe delusions (*P* < 0.05, Bonferroni-corrected) (Fig. 1B). There was no significant FA difference in any white matter region between schizophrenia patients with severe delusions and healthy controls (*P* > 0.05, Bonferroni corrected) (Fig. 1B).

Moreover, voxel-wise whole brain analyses of FA pointed towards the same trend, that is, schizophrenia patients without delusions exhibited decreased FA in multiple white matter regions compared to both patients with severe delusions and healthy controls; however, no significant FA difference was observed between schizophrenia patients with severe delusions and healthy controls (*P* < 0.05, FWE corrected) (Fig. S1).

### GMV differences across groups

**Figure 2:**
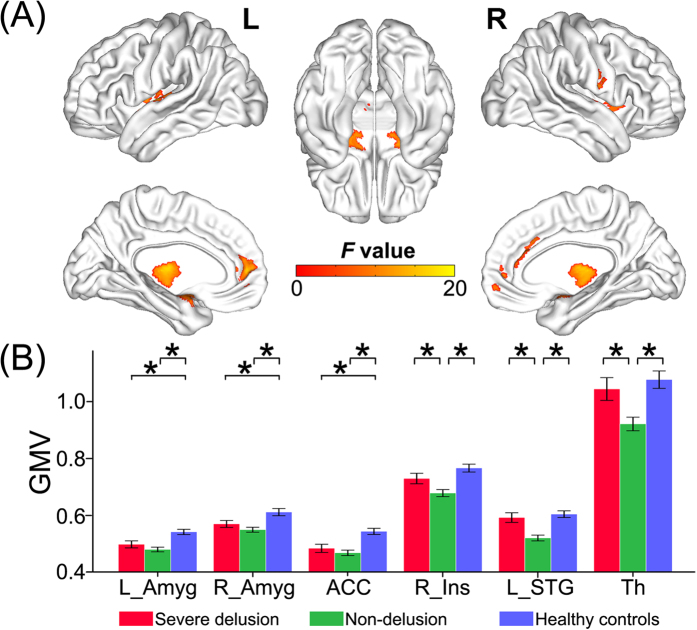
GMV differences across groups. VBM analysis shows gray matter regions with GMV differences (*P* < 0.05, FDR corrected) across the three groups (**A**). A *post hoc* ROI analysis shows the pair-wise comparisons (*P* < 0.05, corrected) (**B**). Error bars indicate the standard error of the mean. *P < 0.05, corrected. Abbreviations: Amyg, amygdala; ACC, anterior cingulate cortex; GMV, gray matter volume; Ins, insula; L, left; R, right; STG, superior temporal gyrus; Th, thalamus.

Six gray matter regions showed significant GMV differences (*P* < 0.05, FDR corrected) among the three groups, including the bilateral amygdala, the bilateral thalamus, the anterior cingulate cortex (ACC), the right insula, and the left superior temporal gyrus (STG) (Fig. 2A, Table S4). The *post hoc* analyses revealed that schizophrenia patients without delusions exhibited significantly reduced GMV in the right insula, the left STG, and the bilateral thalamus compared to both patients with severe delusions and healthy controls (*P* < 0.05, Bonferroni-corrected) (Fig. 2B). There were no significant GMV differences in these brain regions between schizophrenia patients with severe delusions and healthy controls (*P* > 0.05, Bonferroni corrected) (Fig. 2B). Although both patient subgroups showed significantly decreased GMV in the bilateral amygdala and the ACC relative to healthy controls (*P* < 0.05, Bonferroni-corrected), no brain region had a significant GMV difference between the two patient subgroups (*P* > 0.05, Bonferroni-corrected) (Fig. 2B).

Moreover, voxel-wise whole brain analyses of GMV revealed that schizophrenia patients without delusions showed reduced GMV in the left STG compared to patients with severe delusions, and in widespread gray matter regions compared to healthy controls; however, no significant GMV difference was observed between schizophrenia patients with severe delusions and healthy controls (*P* < 0.05, FDR corrected) (Fig. S2).

### CBF differences across groups

**Figure 3:**
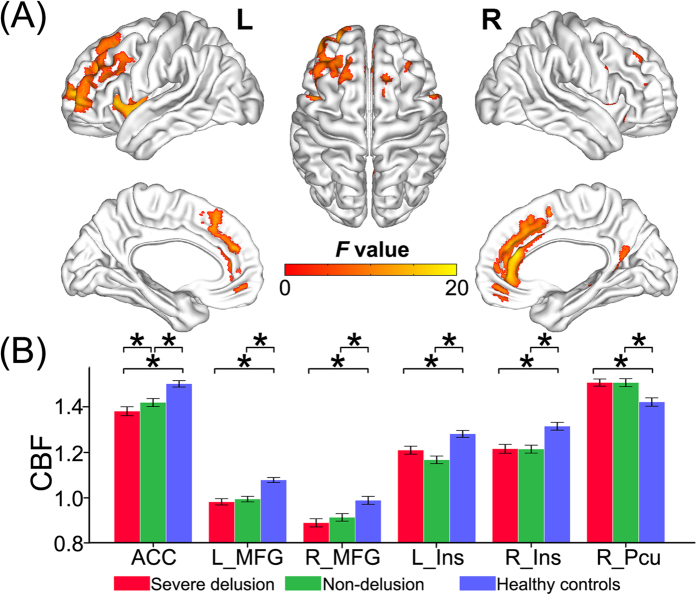
CBF differences across groups. Voxel-based CBF analysis shows gray matter regions with CBF differences (*P* < 0.05, FDR corrected) across the three groups (**A**). A *post hoc* ROI analysis shows the pair-wise comparisons (*P* < 0.05, corrected) (**B**). Error bars indicate the standard error of the mean. *P < 0.05, corrected. Abbreviations: ACC, anterior cingulate cortex; CBF, cerebral blood flow; Ins, insula; L, left; MFG, frontal middle gyrus; Pcu, precuneus; R, right.

Six gray matter regions showed significant CBF differences (*P* < 0.05, FDR corrected) among the three groups including the ACC, the bilateral middle frontal gyrus (MFG) and the bilateral insula, and the right precuneus (Pcu) (Fig. 3A, Table S5). Compared to healthy controls, both patient subgroups showed significantly decreased CBF in the ACC, the bilateral MFG, and the bilateral insula, and significantly increased CBF in the right Pcu (*P* < 0.05, Bonferroni-corrected) (Fig. 3B). There were no significant CBF differences in the bilateral MFG, the bilateral insula, and the right Pcu between the two patient subgroups (*P* > 0.05, Bonferroni corrected) (Fig. 3B). Schizophrenia patients with severe delusions also exhibited significantly decreased CBF in the ACC than patients without delusions (*P* < 0.05, Bonferroni-corrected) (Fig. 3B).

Moreover, voxel-wise whole brain analyses of CBF demonstrated a similar discovery that both patient groups exhibited decreased CBF in multiple gray matter regions compared to healthy controls; however, no significant CBF difference was observed between two patient groups (*P* < 0.05, FDR corrected) (Fig. S3). Notably, although both patient groups showed decreased CBF in the ACC, the spatial extent was much larger in patients with severe delusions than in patients without delusions (Fig. S3).

### FA, GMV and CBF differences without age covariate

The ANCOVA results of FA, GMV and CBF without age covariate are presented in Fig. S4–6. The inter-group differences in FA, GMV and CBF without age covariate were similar to those with age covariate.

### Effects of antipsychotic medications and duration of illness

As shown in Table S6, the results of comparisons between schizophrenia patients with severe delusions and without delusions are very similar before and after controlling for dosage of chlorpromazine equivalents and duration of illness, indicating that the dosage of medications and duration of illness did not significantly influence our results.

### Effects of other symptoms

The results of ROI-based comparisons in FA, GMV and CBF between the two patient groups before and after controlling for the score of PANSS except delusions are shown in Table S7. Although most of the results remained unchanged, some differences (e.g., FA of the BCC, the left ACR and ILF; CBF of the ACC) were not significant after controlling for the score of PANSS except delusions, suggesting that other symptoms may affect the imaging measure differences to some extent.

## Discussion

Using up-to-date imaging techniques (i.e., DKI and 3D-pcASL) and analytical methods (i.e., TBSS and DARTEL), we performed a comprehensive analysis to investigate the neural substrates of delusions in schizophrenia. In contrast to the extensively decreased FA and GMV in schizophrenia patients without delusions, patients with severe delusions did not differ in FA in all white matter regions and in GMV in multiple gray matter regions compared to healthy controls. These findings suggest that schizophrenia patients with severe delusions have nearly normal structural integrity in these regions exhibiting structural impairments in patients without delusions. Both patient subgroups had decreased GMV and altered CBF in several gray matter regions relative to the healthy control group, suggesting that they are common changes in schizophrenia. Schizophrenia patients with severe delusions had further reduced CBF in the ACC than patients without delusions, suggesting that further reduction in the ACC perfusion may be associated with the occurrence of delusions in schizophrenia.

### Common structural and functional alterations in schizophrenia

Compared with the healthy control group, the two patient subgroups consistently showed reduced GMV in the ACC and amygdala, reduced CBF in the MFG and insula, and increased CBF in the right precuneus. However, these measures did not differ between schizophrenia patients with and without delusions. These changes can be considered common changes in schizophrenia, which may be at the root of pathogenesis of this disorder. Structural and functional alterations in these brain regions have been frequently reported in previous studies on schizophrenia. For example, prior studies have revealed gray matter reductions in the ACC and the amygdala in both first-episode and chronic schizophrenia^53^,^54^. The ACC is a crucial region that integrates cognitive and emotional processes in support of goal-directed behaviors^55^,^56^,^57^,^58^ and the amygdala is considered to be the core region in emotional processing^59^. The atrophy in the ACC and amygdala may be related to the difficulty in cognitive and emotional integration in schizophrenia^60^,^61^,^62^. The frontal and insular hypoperfusion is consistent with previous findings from positron emission tomography (PET) and ASL studies^63^,^64^,^65^. The dorsolateral prefrontal cortex (DLPFC) plays an important role in executive control^66^,^67^ and working memory^68^. Perfusion abnormality in the DLPFC appears to account for, at least in part, the cognitive deficits experienced by schizophrenia patients^69^,^70^. The anterior insula is functionally associated with interoception^71^, i.e., the awareness of the body’s internal states consisting of emotional response and complex cognitive state^72^. Given that interoception is associated with perceiving an image of the “self” and make judgments of “self” versus “nonself”^73^, it is reasonable to hypothesize that the disrupted interoception in schizophrenia may be related to the perfusion impairment of the anterior insula^74^. As a core node of the default-mode network (DMN)^75^, the precuneus exhibited increased perfusion in schizophrenia, indicating that high DMN activity may be a neuropathological mechanism of schizophrenia^76^.

### Structural integrity and delusions in schizophrenia

Although schizophrenia patients without delusions had decreased FA in multiple white matter regions and decreased GMV in multiple gray matter regions relative to healthy controls, schizophrenia patients with severe delusions showed no significant decreases in the FA in all white matter regions and in the GMV in most of these gray matter regions relative to healthy controls. That is, schizophrenia patients with severe delusions had nearly normal gray and white matter integrity. Although our findings are in line with several previous studies that reported a positive correlation between structural integrity and delusions in schizophrenia^11^,^12^,^13^,^14^,^15^,^16^,^17^,^18^,^19^,^20^, these findings are inconsistent with other studies that reported a negative correlation between them^21^,^22^,^23^,^24^,^25^,^26^,^27^,^28^,^29^,^30^,^31^,^32^,^33^. The discrepancy across studies may be related to inter-research differences in multiple aspects, such as patient characteristics (subtypes, illness durations, treatments), sample sizes (15–88 cases), imaging techniques (DTI, DKI, diffusion spectrum imaging, and structural MRI), MRI indices (total volume, GMV, white matter volume, gray matter density, white matter density, surface deformity, FA, generalized FA, mean diffusivity, axial diffusivity, radial diffusivity, and probability indices forming part of a bundle of interest), and analytical methods (voxel-wise or ROI-wise analysis, correlation or intergroup comparison). Notably, previous studies have consistently reported that structural and functional alterations of the dorsal medial prefrontal cortex are related to delusions of schizophrenia^15^,^77^, which are in line with our GMV and CBF findings.

Many factors may affect our results. The sample sizes of the three groups are relatively small, which may prevent us from identifying subtle differences between groups. These differences may better account for delusion symptoms. Schizophrenia manifests as a complex composition of many symptoms, including delusions, hallucinations, disorganization, avolition, apathy, and cognitive decline. The heterogeneity of symptoms except for delusions across patients may also affect our results. Moreover, delusions of schizophrenia are not a homogeneous entity and may include multiple subtypes. Because different subtypes of delusions may have different structural substrates in schizophrenia, the heterogeneous subtypes across schizophrenia patients with severe delusions may lower the possibility to find significant results. In addition, although the dosage of medications and duration of illness did not significantly influence our results, we cannot exclude the effects of other factors, such as stage of medications, medication adherence, efficacy or compatibility of medicines.

Regardless of the effects of the above-mentioned factors, the lack of significant structural differences between patients with delusions and healthy controls may be driven by the following mechanisms. First, delusions may be the result of functional impairment (CBF of the ACC: severe delusions < non delusions < controls) rather than structural impairments. Second, there may be subtle delusion-related structural changes that cannot be detected by the current study because of the limit of MRI techniques. Third, the coexistence of structural damage and reorganization in patients with delusions may lower the structural differences between patients with delusions and healthy controls. Finally, the relatively preserved structural integrity may be a prerequisite for the formation of delusions in schizophrenia according to previous studies^12^,^14^,^15^.

### Functional impairments and delusions in schizophrenia

The most important finding of this study is that the CBF in the ACC gradually decreased across the groups, from healthy controls to schizophrenia patients without delusions to patients with severe delusions. This finding suggests that hypoperfusion of the ACC beyond a certain threshold might result in the formation of delusions in schizophrenia, which is consistent with a prior study^78^. The ACC is one of the most important brain regions governing cognitive control. In particular, it has been found to be involved in attention control^79^, conflict monitoring^80^, error monitoring and detection^81^,^82^; deficits in these cognitive functions have been associated with delusion symptoms in schizophrenia^83^,^84^,^85^,^86^,^87^. The ACC is also an important node of the salience network (SN), which serves to identify salient stimuli from the environment and to control cognitive processes in the central executive and default-mode networks^88^,^89^,^90^. In this study, we found reduced CBF in several brain regions (ACC and bilateral insular cortices) of the SN in schizophrenia patients with severe delusions. The hypoperfusion of the SN might impair the process of identifying stimuli as salient, which could then result in a disruption in prediction error that has been associated with the formation of delusions^91^.

### Limitations

Several limitations should be acknowledged in this study. First, most of our patients were chronic schizophrenia and were receiving antipsychotic medications, which may influence our interpretation, i.e., it is difficult to determine if the inter-group differences in imaging measures represent a difference in delusion severity or treatment response. Investigations of medication-naïve first-episode schizophrenia patients may facilitate a more accurate characterization of the schizophrenic delusions. Second, subtypes of schizophrenia (paranoid, disorganized, catatonic, undifferentiated, residual types according to the DSM-IV) and subtypes of delusions (various types according to their intrinsic and extrinsic features^92^) may affect the results of our analysis. However, relatively small sample prevents us from performing analyses for subtypes. Investigations on the neural substrates of certain subtypes of delusions in certain subtypes of schizophrenia may help us better understand the mechanisms underlying delusions in schizophrenia. Third, patients with severe delusions had higher PANSS positive, general and total scores than patients without delusions except the negative score (Table 1), suggesting that the former patients also have more severity in other symptoms besides delusions. The effects of heterogeneity in other symptoms remain unclear and need to be clarified in future studies. Finally, although PANSS is usually considered a standardized scale for schizophrenia symptoms, PANSS P1 is only a rough estimate of delusions and a more refined assessment of delusions should be used.

## Conclusions

Using state-of-the-art multi-modal MRI imaging techniques and analytical approaches, we systematically investigated the structural and functional substrates of delusions in schizophrenia. The results showed that schizophrenia patients with severe delusions had nearly normal FA in all white matter regions and GMV in most of the gray matter regions, suggesting that schizophrenia patients with severe delusions may have relatively normal structural integrity. In addition, schizophrenia patients with severe delusions had further reduced CBF in the ACC than patients without delusions, suggesting that excessively reduced perfusion in the ACC may be associated with the development of delusions in schizophrenia.

## Additional Information

**How to cite this article**: Zhu, J. *et al*. Neural substrates underlying delusions in schizophrenia. *Sci. Rep.* **6**, 33857; doi: 10.1038/srep33857 (2016).

## Supplementary Material

Supplementary Information

## Figures and Tables

**Table 1:** Demographic and clinical characteristics of schizophrenia patients and healthy controls.

Characteristics	Severe delusion (n = 19)	Non-delusion (n = 30)	Healthy controls (n = 30)	*P*-value
Age (years)	34.6 ± 9.4	35.9 ± 9.2	35.3 ± 9.3	0.894^a^
Sex (female/male)	7/12	16/14	15/15	0.512^b^
Antipsychotic dosage (mg/d) (chlorpromazine equivalents)	530.5 ± 514.3	454.0 ± 235.1	NA	0.482^c^
Duration of illness (months)	105.8 ± 102.4	151.6 ±108.5	NA	0.148^c^
PANSS				
Positive score	25.5 ± 7.4	9.9 ± 2.9	NA	<0.001^c^
Negative score	21.4 ± 10.1	17.3 ± 7.5	NA	0.111^c^
General score	39.8 ± 13.3	27.5 ± 7.1	NA	<0.001^c^
Total score	86.7 ± 24.6	54.7 ± 14.3	NA	<0.001^c^

Note. The data are presented as the mean values ± standard deviations. Abbreviations: NA, not applicable; PANSS, The Positive and Negative Syndrome Scale.

^a^One-way ANOVA was used to test the difference in age across the three groups.

^b^Chi-square test was used to test the difference in sex across the three groups.

^c^Two-sample t-test was used to compare the differences in antipsychotic dosage, duration of illness and PANSS scores between two patient groups.
