# Supplementary material for: Neural substrates underlying delusions in schizophrenia
Source: Sci Rep. 2016 Sep 21;6:33857. doi: 10.1038/srep33857 (PMC5030611; doi:10.1038/srep33857)
Supplement: Supplementary Information [file srep33857-s1.doc]

**Full Title:**

**Neural substrates underlying delusions in schizophrenia**

**Jiajia Zhu^1,3^, Chuanjun Zhuo^1,2,3^, Feng Liu^1^, Lixue Xu^1^, Chunshui Yu^1,*^**

**Running Title:**

**Neural substrates of delusions in schizophrenia**

**^1^** Department of Radiology and Tianjin Key Laboratory of Functional Imaging, Tianjin Medical University General Hospital, Tianjin 300052, China

**^2^** Department of Psychiatry Functional Neuroimaging Laboratory, Tianjin Mental Health Center, Tianjin Anding Hospital, Tianjin 300070, China

**^3^** These authors contributed equally to this work.

*** Correspondence to:**

Chunshui Yu. Prof. M.D.

Department of Radiology, Tianjin Medical University General Hospital, No. 154, Anshan Road, Heping District, Tianjin 300052, China.

Phone: +86-22-63062026

Fax: +86-22-63062290

E-mail: [chunshuiyu@tijmu.edu.cn](mailto:chunshuiyu@tijmu.edu.cn)

**Supplemental Materials**

**Table S1.** Association between delusions and white matter abnormalities in schizophrenia patients

| References | Schizophrenia patients | MRI indexes (analyzing methods) | Delusional measure | Main findings |
| --- | --- | --- | --- | --- |
| Wright et al., 1995 | Chronic patients (n=15) | White matter density  (voxel-based correlation) | The sum score of hallucinations and delusions of the SAPS | A negative correlation between the sum score and white matter density in the corpus callosum |
| Szeszko et al., 2008 | Recent onset (< 4.25 years) patients (n=33) | FA  (ROI-based correlation) | Delusion score of the SADS-C + PD | A positive correlation between delusions and FA in the inferior fronto-occipital fasciculus |
| Chan et al., 2010 | First-episode patients (n=39) | FA  (ROI-based correlation) | Delusion score of the PANSS | A positive correlation between delusions and FA in the right temporal-occipital region |
| Makris et al., 2010 | Chronic patients (n=88) | White matter volume  (component-based correlation) | Delusion score of the SAPS | Positive correlations between delusions and volumes in the paralimbic superficial white matter and total sagittal system |
| Whitford et al., 2010 | Chronic patients (n=19) | FA and RD  (ROI-based correlation) | The sum score of delusions and hallucinations of the PANSS | A positive correlation between the sum score and FA and a negative correlation between the sum score and RD in the frontal fibers of corpus callosum |
| Abdul-Rahman et al., 2012 | Chronic patients (n=32) | FA and AD  (ROI-based correlation) | Delusion score of the PANSS | Positive correlations between delusions and leftward asymmetry of FA and AD in the temporal arcuate fasciculus |
| Bracht et al., 2014 | Chronic patients (n=24) | PIBI-values  (ROI-based correlation) | The sum score of delusions and hallucinations of the PANSS | A positive correlation between the sum score and PIBI of the left amygdala-nucleus accumbens connection |
| Fitzsimmons et al., 2014 | First-episode patients (n=18) | MD and RD  (ROI-based correlation) | Delusion score of the SAPS | Negative correlations between delusions and MD and RD in the cingulum bundle |
| Whitford et al., 2014 | Chronic patients with (n=10) and without (n=13) delusions | FA  (ROI-based comparison) | Delusion score of the SAPS | Patients with delusions exhibited lower FA in 4 large fibers than healthy controls and patients without delusions |
| Whitford et al., 2014 | Chronic patients (n=24) | FA  (ROI-based correlation) | Delusion score of the SAPS | A negative correlation between delusions and FA in the anterior part of the cingulum bundle |
| Wu et al., 2014 | Chronic patients (n=18) | GFA  (ROI-based correlation) | The sum score of delusions and hallucinations of the PANSS | A negative correlation between the sum score and GFA in the right dorsal language pathway |
| Son et al., 2014 | Chronic patients (n=43) | FA  (voxel-based correlation) | Peters Delusions Inventory | A negative correlation between delusions and FA in a widespread white matter area |

Abbreviations: AD, axial diffusivity; FA, fractional anisotropy; GFA, generalized fractional anisotropy; PIBI, probability indices forming part of a bundle of interest; RD, radial diffusivity; PANSS, The Positive and Negative Syndrome Scale; ROI, region of interest; SADS-C + PD, Schedule for Affective Disorders and Schizophrenia—change version with psychosis and disorganization items; and SAPS, Scale for Assessment of Positive Symptoms.

**Table S2.** Association between delusions and gray matter changes in schizophrenia patients

| References | Schizophrenia patients | MRI indexes (analyzing methods) | Delusional measure | Main findings |
| --- | --- | --- | --- | --- |
| Menon et al., 1995 | Chronic patients (n=20) | Total volume  (ROI-based correlation) | Delusion score of the SAPS | A positive correlation between delusions and total volume in the left posterior superior temporal gyrus |
| Wright et al., 1995 | Chronic patients (n=15) | Gray matter density  (voxel-wise analysis) | The sum score of hallucinations and delusions of the SAPS | A negative correlation between the sum score and gray matter density in the left superior temporal lobe |
| Szeszko et al., 1999 | First-episode patients (n=19) | Total volume  (ROI-based correlation) | Delusion score of the SADS-C + PD | A positive correlation between delusions and ratio of right orbital frontal to right 'archicortical' volume |
| Maruff et al., 2005 | Chronic patients with (n=12) and without (n=11) delusions | GMV  (voxel-based comparison) | Passivity delusions | The passivity delusion group had reduced GMV in the parietal and frontal cortices than the non-delusion group |
| Takahashi et al., 2006 | Chronic patients (n=65) | GMV  (ROI-based correlation) | Delusion score of the SAPS | A negative correlation between delusions and GMV in the superior temporal gyrus |
| Mamah et al., 2007 | Chronic patients (n=54) | Total volume  (ROI-based correlation) | Delusion score of the SAPS | A negative correlation between delusions and nucleus accumbens volume |
| Yamasaki et al., 2007 | Chronic patients (n=17) | GMV  (ROI-based correlation) | Delusion-related sum score of the PANSS | A negative correlation between delusion behaviour and the right planum temporale volume |
| Whitford et al., 2009 | First-episode patients (n=31) | GMV  (voxel-wise analysis) | Delusion score of the PANSS | A positive correlation between delusions and GMV in the dorso-medial frontal cortex |
| Cascella et al., 2011 | Chronic patients (n=43) | GMV  (voxel-wise analysis) | Delusion score of the SAPS | Negative correlations between delusions and GMV of the left claustrum and right insula |
| Palaniyappan et al., 2011 | Chronic patients (n=57) | GMV  (voxel-wise analysis) | The sum score of hallucinations and delusions of the SSPI | Negative correlations between the sum score and GMV in the regions of the salience network |
| Spalletta et al., 2013 | Chronic patients with (n=28) and without (n=47) delusions | GMV  (voxel-wise analysis) | Somatic delusions of the SAPS | Patients with delusions had reduced GMV in the left fronto-insular cortex than healthy controls and patients without delusions |
| Zierhut et al., 2013 | Chronic patients (n=32) | Surface deformity | Delusion score of the PANSS | A negative correlation between delusions and the hippocampal surface deformity in the CA1 subfield |

Abbreviations: GMV, gray matter volume; PANSS, The Positive and Negative Syndrome Scale; SADS-C + PD, Schedule for Affective Disorders and Schizophrenia—change version with psychosis and disorganization items; ROI, region of interest; SAPS, Scale for Assessment of Positive Symptoms; and SSPI, Symptoms and Signs in Psychotic Illness.

**Table S3.** Subtypes of delusions in 19 schizophrenia patients with severe delusions

| Subjects | Persecutory | Jealous | Guilt | Grandiose | Religious | Somatic | Reference | Control | Mindreading |
| --- | --- | --- | --- | --- | --- | --- | --- | --- | --- |
| Patient01 | **√** |  |  |  |  | **√** | **√** | **√** |  |
| Patient02 | **√** |  | **√** | **√** |  |  | **√** | **√** |  |
| Patient03 | **√** |  |  | **√** |  | **√** | **√** | **√** |  |
| Patient04 |  |  |  | **√** | **√** |  | **√** | **√** | **√** |
| Patient05 |  |  |  |  |  | **√** |  | **√** |  |
| Patient06 | **√** |  |  | **√** |  |  | **√** |  | **√** |
| Patient07 | **√** |  |  |  |  | **√** | **√** |  |  |
| Patient08 | **√** |  |  | **√** | **√** |  | **√** | **√** |  |
| Patient09 | **√** |  |  |  |  |  | **√** | **√** | **√** |
| Patient10 | **√** |  |  |  |  |  | **√** |  | **√** |
| Patient11 | **√** |  |  |  |  |  | **√** | **√** | **√** |
| Patient12 | **√** |  |  |  |  |  |  |  |  |
| Patient13 |  |  | **√** | **√** |  |  |  |  | **√** |
| Patient14 | **√** |  |  |  |  |  |  |  |  |
| Patient15 | **√** |  |  |  |  |  | **√** | **√** |  |
| Patient16 |  |  |  |  |  |  |  | **√** |  |
| Patient17 | **√** |  |  |  |  | **√** |  | **√** |  |
| Patient18 | **√** |  |  | **√** | **√** |  | **√** | **√** | **√** |
| Patient19 | **√** |  |  |  |  |  |  |  |  |

Note. Most schizophrenia patients exhibited more than one subtype of delusions.

**Table S4.** Brain areas with significant differences in GMV across the three groups

| Regions | BA | Cluster size (voxels) | Peak *F*-score | MNI coordination  (x, y, z) |
| --- | --- | --- | --- | --- |
| L_Amyg |  | 404 | 15.15 | -18, 0, -18 |
| R_Amyg |  | 104 | 12.27 | 21, 1.5, -16.5 |
| ACC | 32 | 1163 | 18.28 | 0, 51, 6 |
| R_Ins | 13 | 458 | 12.09 | 40.5, 7.5, 1.5 |
| L_STG | 22 | 948 | 20.53 | -51, 0, -1.5 |
| Th |  | 1307 | 15.69 | 0, -18, 9 |

Abbreviations: BA, Brodmann’s area; MNI, Montreal Neurological Institute; Amyg, amygdala; ACC, anterior cingulate cortex; Ins, insula; STG, superior temporal gyrus; Th, thalamus; L, left; R, right.

**Table S5.** Brain areas with significant differences in CBF across the three groups

| Regions | BA | Cluster size (voxels) | Peak *F*-score | MNI coordination  (x, y, z) |
| --- | --- | --- | --- | --- |
| ACC | 32 | 2789 | 19.97 | 4, 36, 22 |
| L_MFG | 9, 10 | 2512 | 15.03 | -28, 58, 6 |
| R_MFG | 10,46 | 191 | 9.80 | 46, 46, 12 |
| L_Ins | 13 | 1053 | 18.33 | -40, 10, -10 |
| R_Ins | 13 | 442 | 15.76 | 48, 6, -6 |
| R_Pcu | 23 | 108 | 8.86 | 6, -58, 16 |

Abbreviations: BA, Brodmann’s area; MNI, Montreal Neurological Institute; ACC, anterior cingulated cortex; MFG, frontal middle gyrus; Ins, insula; Pcu, precuneus; L, left; R, right.

**Table S6.** Imaging measure differences between schizophrenia patients with severe delusions and without delusions before and after controlling for dosage of chlorpromazine equivalents and duration of illness

| Measure-ROI | Before control | After control |
| --- | --- | --- |
| FA-L_ACR | 7.576(0.008)* | 6.332(0.016)* |
| FA-R_ACR | 6.848(0.012)* | 6.982(0.011)* |
| FA-BCC | 5.783(0.020)* | 4.824(0.034)* |
| FA-GCC | 6.282(0.016)* | 5.940(0.019)* |
| FA-L_ILF | 10.099(0.003)* | 8.130(0.007)* |
| FA-R_ILF | 8.904(0.005)* | 8.922(0.005)* |
| FA-L_OR | 20.224(<0.001)* | 20.658(<0.001)* |
| FA-R_OR | 21.590(<0.001)* | 19.125(<0.001)* |
| FA-SCC | 7.762(0.008)* | 6.581(0.014)* |
| FA-R_SCR | 3.331(0.075) | 2.620(0.113) |
| GMV-L_Amyg | 2.039(0.160) | 1.792(0.188) |
| GMV-R_Amyg | 3.805(0.057) | 3.816(0.057) |
| GMV-ACC | 1.356(0.250) | 1.138(0.292) |
| GMV-R_Ins | 7.553(0.009)* | 7.286(0.010)* |
| GMV-L_STG | 18.989(<0.001)* | 16.949(<0.001)* |
| GMV-Th | 18.677(<0.001)* | 17.055(<0.001)* |
| CBF-ACC | 5.239(0.027)* | 6.870(0.012)* |
| CBF-L_MFG | 0.864(0.358) | 1.044(0.313) |
| CBF-R_MFG | 0.806(0.374) | 1.472(0.232) |
| CBF-L_Ins | 1.524(0.223) | 1.354(0.251) |
| CBF-R_Ins | 0.163(0.688) | 0.683(0.413) |
| CBF-R_Pcu | 0.114(0.737) | 0.336(0.565) |

Note. The data are shown as the *F* value (*P* value). * *P* < 0.05. Abbreviation: ACC, anterior cingulate cortex; ACR, anterior corona radiata; Amyg, amygdala; BCC, body of corpus callosum; CBF, cerebral blood flow; FA, fractional anisotropy; GCC, genu of corpus callosum; GMV, gray matter volume; ILF, inferior longitudinal fasciculus; Ins, insula; L, left; MFG, frontal middle gyrus; OR, optic radiation; Pcu, precuneus; R, right; SCC, splenium of corpus callosum; SCR, superior corona radiata; STG, superior temporal gyrus; Th, thalamus.

**Table S7.** Imaging measure differences between schizophrenia patients with severe delusions and without delusions before and after controlling for the score of PANSS except delusions

| Measure-ROI | Before control | After control |
| --- | --- | --- |
| FA-L_ACR | 7.576(0.008)* | 4.028(0.051) |
| FA-R_ACR | 6.848(0.012)* | 4.449(0.041)* |
| FA-BCC | 5.783(0.020)* | 4.003(0.052) |
| FA-GCC | 6.282(0.016)* | 4.254(0.045)* |
| FA-L_ILF | 10.099(0.003)* | 1.805(0.186) |
| FA-R_ILF | 8.904(0.005)* | 5.202(0.027)* |
| FA-L_OR | 20.224(<0.001)* | 8.088(0.007)* |
| FA-R_OR | 21.590(<0.001)* | 14.102(0.001)* |
| FA-SCC | 7.762(0.008)* | 4.366(0.042)* |
| FA-R_SCR | 3.331(0.075) | 2.989(0.091) |
| GMV-L_Amyg | 2.039(0.160) | 2.067(0.158) |
| GMV-R_Amyg | 3.805(0.057) | 2.010(0.163) |
| GMV-ACC | 1.356(0.250) | 1.148(0.290) |
| GMV-R_Ins | 7.553(0.009)* | 13.186(0.001)* |
| GMV-L_STG | 18.989(<0.001)* | 14.189(<0.001)* |
| GMV-Th | 18.677(<0.001)* | 7.459(0.009)* |
| CBF-ACC | 5.239(0.027)* | 1.539(0.221) |
| CBF-L_MFG | 0.864(0.358) | 1.271(0.266) |
| CBF-R_MFG | 0.806(0.374) | 0.256(0.616) |
| CBF-L_Ins | 1.524(0.223) | 0.414(0.523) |
| CBF-R_Ins | 0.163(0.688) | 0.376(0.543) |
| CBF-R_Pcu | 0.114(0.737) | 0.116(0.735) |

Note. The data are shown as the *F* value (*P* value). * *P* < 0.05. Abbreviation: ACC, anterior cingulate cortex; ACR, anterior corona radiata; Amyg, amygdala; BCC, body of corpus callosum; CBF, cerebral blood flow; FA, fractional anisotropy; GCC, genu of corpus callosum; GMV, gray matter volume; ILF, inferior longitudinal fasciculus; Ins, insula; L, left; MFG, frontal middle gyrus; OR, optic radiation; PANSS, The Positive and Negative Syndrome Scale; Pcu, precuneus; R, right; SCC, splenium of corpus callosum; SCR, superior corona radiata; STG, superior temporal gyrus; Th, thalamus.


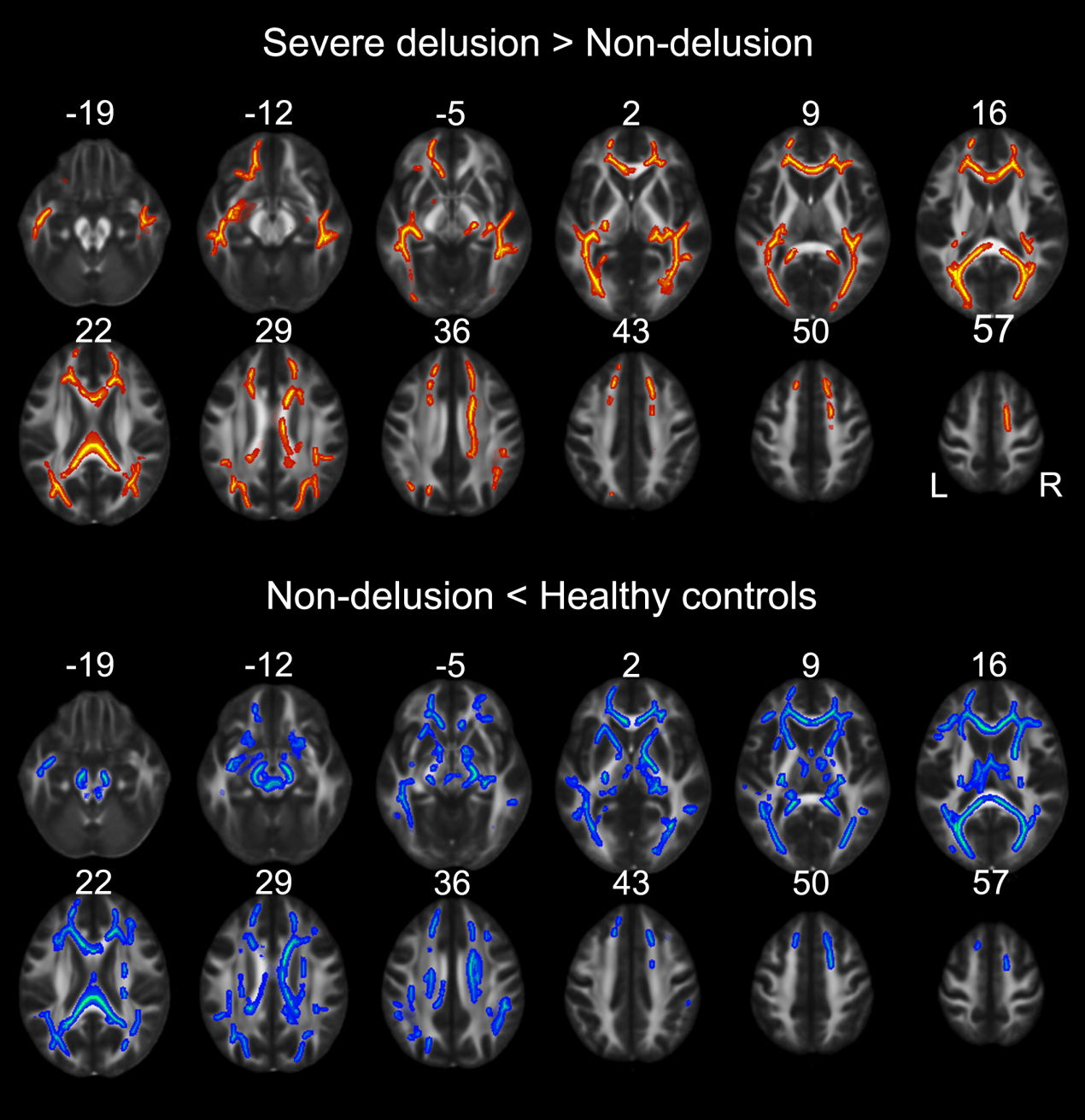


Figure S1. Voxel-wise whole brain comparisons in FA between every two of the three groups. Abbreviations: L, left; R, right.


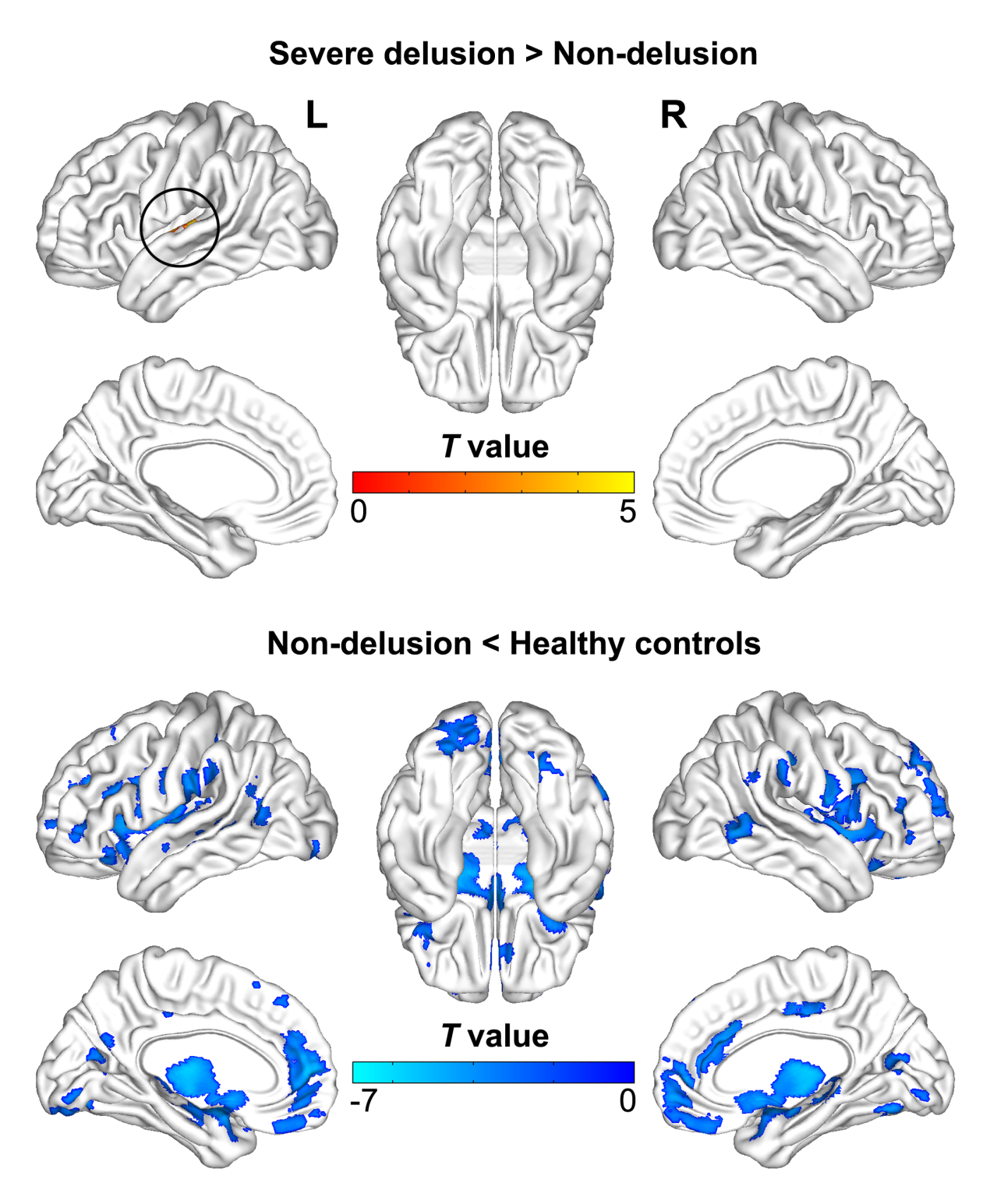


Figure S2. Voxel-wise whole brain comparisons in GMV between every two of the three groups. Abbreviations: L, left; R, right.


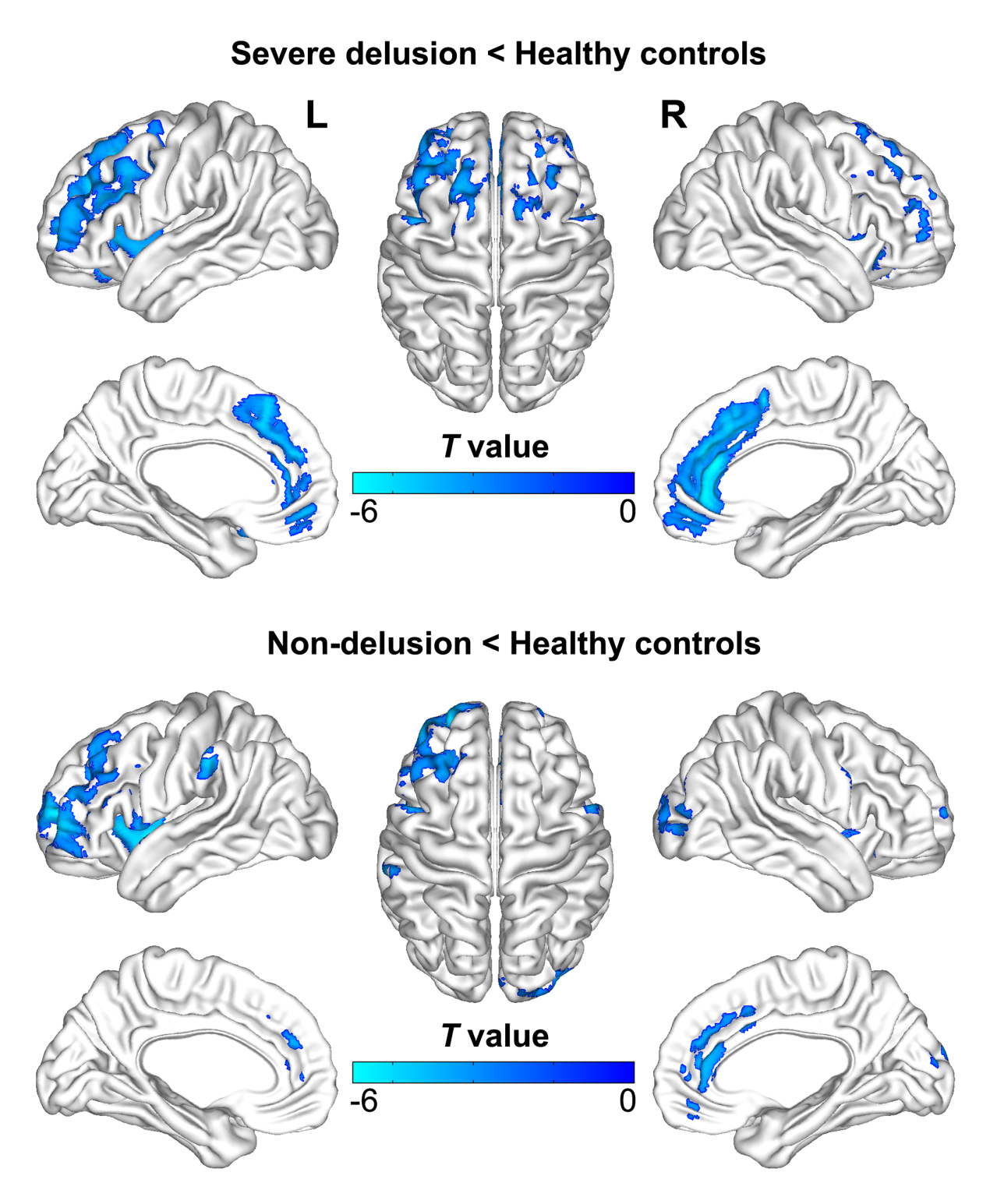


Figure S3. Voxel-wise whole brain comparisons in CBF between every two of the three groups. Abbreviations: L, left; R, right.


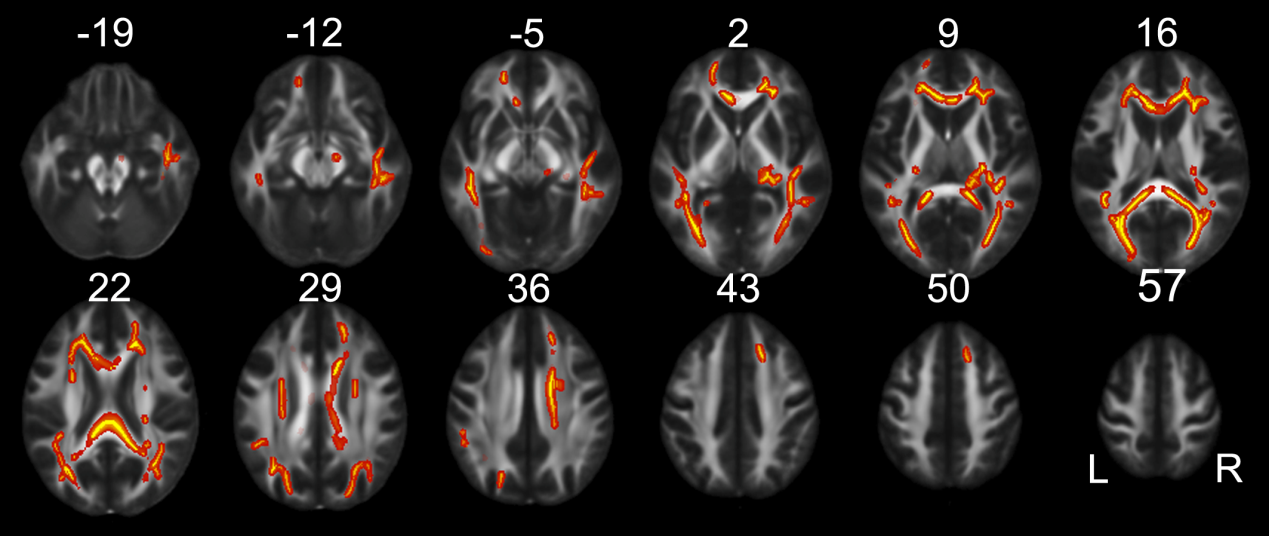
Figure S4. FA differences across groups without age covariate. TBSS analysis shows white matter tracts with FA differences (*P* < 0.05, FWE corrected) across the three groups. Abbreviations: L, left; R, right.


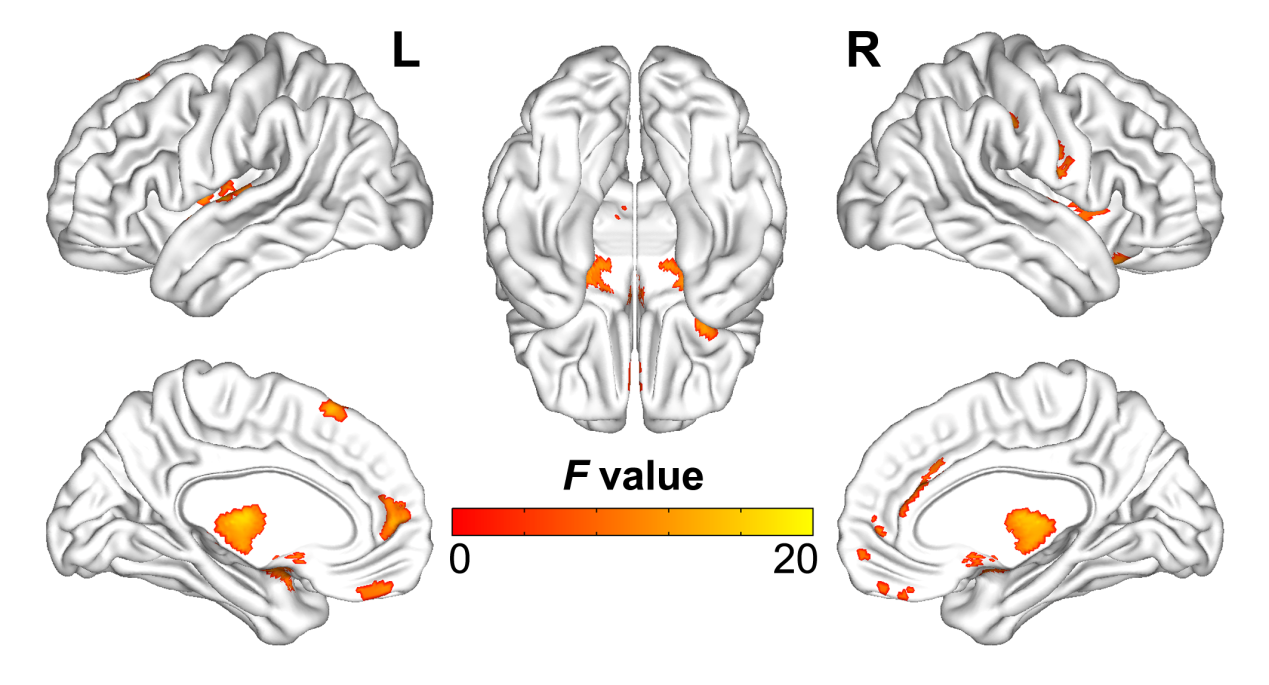


Figure S5. GMV differences across groups without age covariate. VBM analysis shows gray matter regions with GMV differences (*P* < 0.05, FDR corrected) across the three groups. Abbreviations: L, left; R, right.


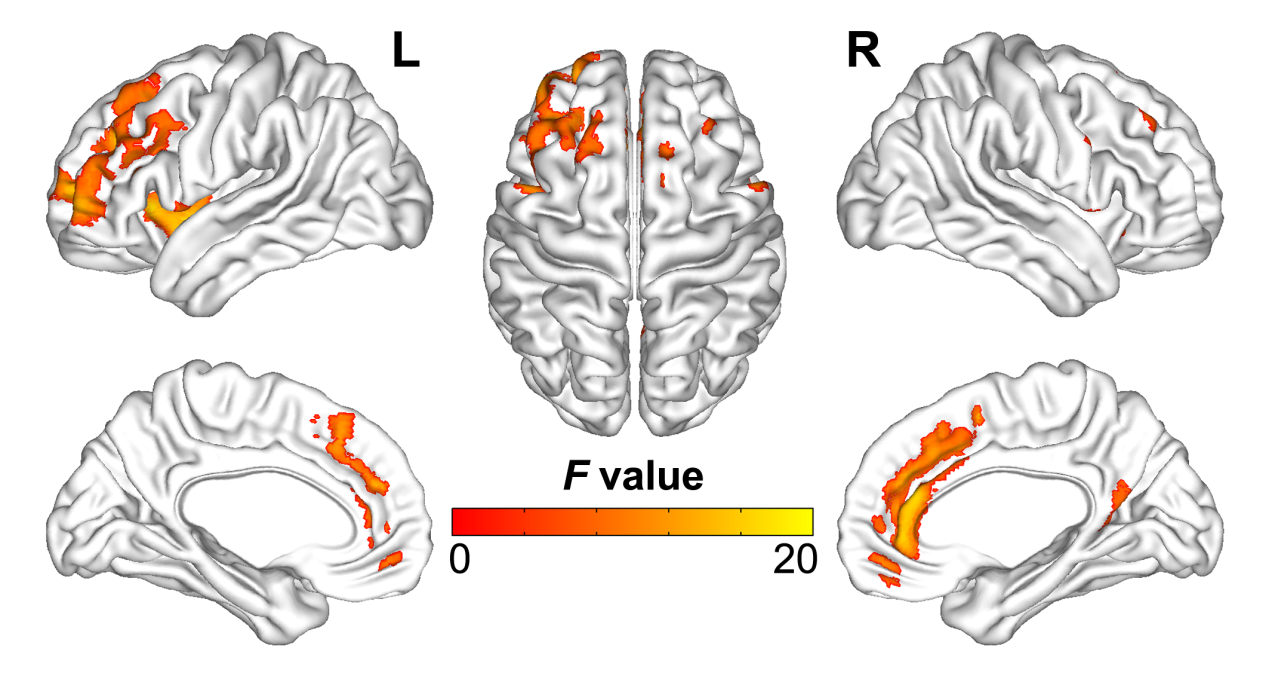
Figure S6. CBF differences across groups without age covariate. Voxel-based CBF analysis shows gray matter regions with CBF differences (*P* < 0.05, FDR corrected) across the three groups. Abbreviations: L, left; R, right.
